# An integrative model of patients’ perceived value of healthcare service quality in North Cyprus

**DOI:** 10.1186/s13690-021-00738-6

**Published:** 2021-12-20

**Authors:** Mert Sanıl, Fehiman Eminer

**Affiliations:** 1 Faculty of Health Sciences, European University of Lefke, Gemikonagı-Lefke, North Cyprus, TR-10 Mersin, Turkey; 2 Faculty of Economics and Administrative Sciences, European University of Lefke, Gemikonagı-Lefke, North Cyprus, TR-10 Mersin, Turkey

**Keywords:** Affordability, Accessibility, Acceptability, Perceived healthcare service value, North Cyprus

## Abstract

**Background:**

Improving healthcare quality has become an essential objective for all health institutions worldwide to address the need to improve services, manage costs and satisfy patient expectations about the quality of care. As health is one of the leading service sectors of the North Cyprus economy, analysing patients’ perceived value of healthcare service quality is crucial. In this research, a comparative analysis of existing models revealed affordability, acceptability and accessibility as the leading modern service quality indicators affecting patients’ perceived value of healthcare service quality. The quality of services is a leading factor impacting business competition and retention dictated by the current market. This study aimed to investigate the factors that influence patient perceptions of healthcare service quality in North Cyprus.

**Methods:**

A self-administered questionnaire was carried out among 388 patients of public and private hospitals in North Cyprus, and the data were analysed using partial least squares-structural equation modelling.

**Results:**

Empirical results highlight that the acceptability of healthcare services is a prerequisite for perceiving a high value of service quality. The affordability and accessibility of services, respectively, were less effective. Results concerning mediating effects confirm that acceptability could fully mediate the relationship between affordability and perceived value and could partially mediate the impact of accessibility on the perceived quality of healthcare services.

**Conclusion:**

This study contributes to healthcare theory and practice by developing a conceptual framework to provide policymakers and managers with a practical understanding of factors that affect healthcare service quality.

## Background

Considering global competition and openness to external markets, it has also become essential for health organizations to adopt a “quality improvement” strategy that guarantees high-quality services [1, 2]. The survivability of these organizations relies heavily on consumers using their services and deciding that the services meet their immediate requirements [3]. Parasuraman et al. [4] noted that service quality is one of the leading factors affecting business competition and retention dictated by the current market. Service quality, therefore, is considered a key indicator of customer attraction and loyalty. These offer additional business advantages, such as positive word-of-mouth, improved customer retention and happy and motivated staff, which can, in turn, increase profit margins and reduce advertisement costs and, most importantly, increase customer satisfaction and enlarge market shares [5, 6]. Greater profitability and improved financial performance lead to consumer satisfaction, causing expanding market shares. Unsurprisingly, therefore, this enduring circle of service quality has caught the eye of many market researchers in the service sector. Managers must foster a suitable, efficient and competitive market and create operational strategies to understand the delivery of organizational service quality [7].

Healthcare provision relies on a very special coordinated grouping of consultants, doctors, nurses and non-nursing specialists. Equally important are hospitals, clinics and nursing homes and their geographical locations. The customer, client or patient will receive or require the available services for their health requirement, which will include stipulated fees. The obvious balance is an acceptable charge for the commensurate service, and this must be understood by both the customer and provider. Such a mutual and informed understanding of the service and cost also causes an acute awareness of the proper level of provision for the healthcare industry, where it becomes more challenging to satisfy a customer (patient). In a situation like this, it is necessary to understand that one of the critical factors of satisfying patients in a hospital is service quality [8].

Patients’ assessments of healthcare services are subjective, as it is not easy for recipients to describe the quality of these services; thus, there is little or no precise expectation of the service quality. The patient’s evaluation can only be a benchmark of a perceived quality-based outcome instead of that of an objective item. Healthcare service quality is also intangible owing to the diversity of offered services. Delivery and improvement in health are simultaneous functions [9]. According to Lewis [10], perceived service quality is a consumer judgment that occurs from comparing consumers’ expectations from services with their perceptions of actual service performance.

The medical pattern and old routine of service quality are being abandoned in this new age of medical care and repair, where the consumer is aware of the improving standards of medical management towards a more scientific and helpful mode [8].

Therefore, conventional performance indicators are no longer sufficient for healthcare institutions. Limited resources are a major cause of the increased interest in modern performance measurement in healthcare. The current methods contain measures linked to costs and those associated with patient satisfaction and quality [11]. Measuring the quality of healthcare services from the patient’s point of view is a crucial factor in evaluating the performance of this important sector because patients provide correct information (feedback) that truly reflects the performance of health institutions [6, 12].

Improving healthcare quality has become an important objective for all health systems and organizations worldwide to address the need to improve poor health services, manage costs and meet increasing patient expectations about the quality of care and healthcare services [1]. Interestingly, the healthcare sector continues to grow and thrive in the Middle East. Despite having seen positive investments in infrastructure and services in Middle Eastern countries during recent years, unfortunately, the region continues to grapple with issues around pricing and access, affordability and regulation of medicines [13]. Like many countries in the region, North Cyprus is now concerned about providing cost-effective and high-quality healthcare services. Since North Cyprus restructured its healthcare system a decade ago, the number of hospitals and their bed capacity have significantly increased; therefore, focusing on healthcare service quality has become even more critical [1, 14].

In North Cyprus, the healthcare industry is going through a transition period where the future may well see significant and challenging changes both in the provision of healthcare and its providers [15]. As North Cyprus is a small island with a population of only approximately 400,000, the service sector was chosen as a leading sector for economic development. The health sector is one of the leading service sectors of the North Cyprus economy. This can explain the main reason for the growing number of private healthcare providers, as there is increasing demand not only from the local people but also from other countries. Its strategic location in the cross section of Asia, Europe and Africa attracts many people from other countries, such as Turkey and European and African countries.

People living in North Cyprus can access healthcare via two main care pathways – that is, public and private healthcare pathways. In the public health system, care is provided for free or at discounted prices to those with social security insurance, which is required for everyone in the workforce if they are employed by the government or in the private sector, or if they are self-employed. Care in accident and emergency departments is free for everyone. However, few citizens in North Cyprus use the public healthcare system to access higher quality services [16]. As demand for healthcare grows, like in other countries, the increase in demand for private health services outpaces that for public health services. This reality has changed the structure of private providers, from small clinics to small and large hospitals. Out-of-pocket payment is the primary financing method for those private hospitals. Rahmioglu et al. [16] explained that individuals had purchased voluntary health insurance in recent years, but this is not yet widespread.

Consequently, demand for healthcare increases, and private hospitals benefit from this. However, this does not mean that there will be demand and willingness to pay for the healthcare services provided by private hospitals without quality expectations. As the number of hospitals increases, the competition forces healthcare providers to increase quality and lower prices. As no study has examined whether this theory works for North Cyprus, it is difficult to make a comment. However, there are alternative markets, and if patients are not satisfied, they can easily find alternatives to meet their healthcare needs. Turkey and South Cyprus are two main alternatives for people from North Cyprus to easily buy healthcare services. The North Cyprus health system also lacks a reliable instrument to systematically study customer perceptions of service quality, which hinders their ability to continuously achieve and improve business excellence. Therefore, it is vital to analyse patients’ perceived value of healthcare service quality in North Cyprus [17]. The present study addressed this gap.

Health coverage is one of the most important factors that affect the development of countries. There are attempts at the international level, such as the efforts of the World Health Organization (WHO), to provide universal health coverage. With the recent COVID-19 pandemic, the world has been experiencing dramatic years that have made it apparent that healthcare should be universal. It is not possible to limit health coverage and foster a healthy society. WHO promotes universal health coverage (UHC) internationally. Countries have health policies to cover everyone regardless of their economic or social status. UHC aims to provide a system that benefits everyone by giving them access to the healthcare services they need without considering differences in income. This must be supported with high-quality health services for everyone [18]. Affordability, insurance coverage, accessibility and healthcare services are considered important factors for universal coverage. As North Cyprus is not a WHO member, universal coverage is not under the consideration of its political bodies. However, as mentioned above, there is a public provision of healthcare services, and households under government social security coverage can benefit. Although this provision offers healthcare coverage to everyone, it does not give any indication of the quality, accessibility and availability of the healthcare services. Although the constitution of North Cyprus accords equal rights to everyone to benefit from public healthcare services, it is not applicable because there is a high share of out-of-pocket payment for health services in North Cyprus [16, 19].

Not being a member of WHO is not sufficient for justifying the high share of out-of-pocket payments in North Cyprus, but it must be considered as one of the important reasons. The country’s health policy should be built on WHO’s international standards for improving healthcare services, since WHO has the core global functions of establishing, monitoring and boosting international norms and standards and coordinating multiple actors (countries and multilateral institutions) towards the common goal of universal health coverage. In this manner, even North Cyprus would be able to minimize external costs and improve health service coverage and the quality of healthcare. As there is no study on this subject, it is difficult to expect changes in health policy towards international standards. Academic studies would help to create awareness and force policymakers to improve the healthcare system.

This study aimed, first, to empirically investigate the factors influencing patients’ perception of quality within the North Cyprus healthcare sector. Second, the study was intended to contribute to the literature on general customer expectations within the North Cyprus healthcare industry. Finally, the study set out to yield pertinent recommendations for both public and private policymakers in North Cyprus.

The rest of this paper is structured as follows. The next section reviews previous studies and investigations relating to perceived service quality and factors including affordability, accessibility and acceptability. It also presents the research hypotheses. The third section explains the research method, including measurement, sample and data collection techniques. Additional empirical results from the questionnaire are analysed, presented and discussed in the fourth section. The final section contains the discussion, implications, limitations and conclusion of the study.

## Literature review

### Healthcare service quality

As discussed, the quality of healthcare is one of the most important topics in the health service sector today. Due to its intangible characteristics, no single universally accepted definition exists. Healthcare service quality is even more difficult to define and measure than in other sectors [20]. It is often exceedingly difficult to reproduce consistent healthcare services; among the plethora of practitioners, managers, places, times and customers, they can differ vastly. Quality standards are more challenging to establish for in-service operations, reinforcing that professionals in the healthcare sector supply and orchestrate services differently because of their levels of training, discipline experience and individual abilities, attitudes and personalities [21, 22]. Thus, one is unlikely to be able to judge the level of quality before receiving the service. Unlike manufactured goods, it is less probable to have a final quality check of healthcare services. Therefore, healthcare outcomes cannot be guaranteed. Unlike manufactured goods, a healthcare service or product cannot be touched, manipulated, viewed, counted or measured [22, 23].

Mosadeghrad [24] defined quality healthcare as “consistently delighting the patient by providing efficacious, effective and efficient healthcare services according to the latest clinical guidelines and standards, which meet the patients’ needs and satisfy providers.”

According to Crow et al. [25], some attributes integrate to affect the quality of care rendered. Measurements of the overall performance of healthcare delivery might incorporate input details, process and outcomes. Although there is much debate over how the quality of care should be assessed, the level of patient satisfaction is a highly relevant signal because it shows patients’ perceptions of the standards achieved, their evaluations of how good the care was and the providers’ success in meeting clients’ values and expectations.

To develop the conceptualized model, this study attempted to identify the most effective dimensions commonly used by significant studies to assess patients’ perceived value of healthcare service quality.

In Lim and Tang’s [9] study aimed at identifying patients’ expectations and perceptions of Singapore hospitals’ service quality, besides the five traditional dimensions of tangibility, reliability, responsiveness, assurance and empathy used by Youssef et al., they added the two important dimensions of accessibility and affordability to the final research model.

Sovd et al. mentioned that accessibility, acceptability and equity are three dimensions that define WHO’s conception of healthcare service quality. They developed their research model according to the WHO dimension of quality and included variables conforming to accessibility and acceptability, which enabled a unique analysis predicting customer satisfaction by comparing the perceived importance of diverse quality scopes. The dimension of equity, however, was not assessed in their study.

According to the research framework of Fatima et al.’s study on hospital healthcare service quality, patient satisfaction and loyalty showed that in private hospitals, providing a clean and efficient environment, effective communication, accessible and convenient services and an advanced input system are the most significant aspects of patients’ perceived healthcare service quality.

Oliver and Mossialos [27] paid great attention to equity of access to healthcare services. Based on their fundamental research, equal access to equal needs entails conditions whereby those with equal needs have equal opportunities to access healthcare. Regarding different acceptable motives, those in equal need and with equal opportunities to access healthcare services might not have equal use of these opportunities. They concentrated mostly on clusters defined by income, geographical residence and (to a smaller extent) ethnicity.

In Baltussen et al.’s [28] research in Burkina Faso, enhancing health services and medicines and availability and accessibility were introduced as the two main health policy actions that must be taken.

Penchansky and Thomas [29] proposed a taxonomic definition of access as a significant concept of health policy. This concept includes five reasonably distinct dimensions: availability, accessibility, affordability, accommodation and acceptability.

Cheng et al. [30] discussed hospitals’ quality competition in Taiwan’s healthcare market and investigated what quality and cost factors influence patients’ perceptions of healthcare services. Their results disclosed that out-of-pocket cost was a significant predictor of perceived expensiveness. This finding also revealed that a perceived expensive price might affect a customer’s hospital recommendation, meaning that a patient may not trust that the service is worth the price.

The outcome variable for assessing customer-perceived service quality in healthcare organizations in D’Souza and Sequeira’s [8] study included five dimensions: user-friendliness, accessibility, privacy and confidentiality, comprehensiveness of testing and comprehensiveness of treatment.

Niëns and Brouwer [31] and Niëns et al. [32] studied the importance and challenges of measuring the affordability of healthcare services and medicines on the health policy agenda in Indonesia.

Dillip et al.’s [33] study focused on acceptability as a neglected dimension of healthcare service quality perceived by patients. Based on the outcomes of their analysis conducted in Tanzania, they found that the social acceptability of healthcare services is fundamental to ensure successful control and management of health problems in society.

Dansereau et al. [34] conducted another effective study considering patient satisfaction and perceived healthcare quality in Zambia. The conceptualized model of the study included five independent variables: health personnel practice and conduct, accessibility of care, cost of care, adequacy of resources and services, and healthcare delivery.

Li et al. [35] compared perceptions of the quality of primary care in China. Their research model proposed using primary care attributes – comprising accessibility, continuity, coordination of services and information, comprehensiveness, service availability and provision – as indicators to monitor primary care systems.

Sekhon et al. [36] observed that acceptability had become a key consideration in the design, evaluation and implementation of healthcare service perceptions through their developed theoretical framework. Their findings evinced that despite frequent claims that accessibility is assessed in determining healthcare service quality, it is evident that acceptability research could be more robust.

In an interesting research paper titled “The acceptability of healthcare: from satisfaction to trust,” Dyer et al. [37] explained that the assessment of healthcare quality increasingly emphasizes lay acceptability, as evidenced by the emergence of patient satisfaction and patient-centered care in the literature and the policy.

Cinaroglu and Baser’s [11] study showed that healthcare institutions face significant challenges in implementing quality initiatives, such as performance measurement. In a study on 81 provinces in Turkey using a path analytic model, the authors explored the accessibility of services and utilization as the two main dimensions of performance measurement. These factors have the potential to enhance healthcare service outcomes, performance and quality.

Further, Maxwell [38] identified six dimensions of quality – effectiveness, acceptability, efficiency, access, equity and relevance – while Camilleri and O’Callaghan [39] considered seven attributes for measuring the quality of hospital services: professional and technical care, service personalization, price, environment, patient amenities, accessibility and catering. Thus, the aforementioned studies showed that patients’ perceived quality of healthcare services can be assessed according to different dimensions, such as availability, accessibility, affordability, acceptability, environment, effective communication, privacy, confidentiality, caring, responsiveness, amenities and facilities. However, a comparative analysis shows that the affordability, acceptability and accessibility of healthcare services are the most effective indicators and the minimum number that should be used to assess healthcare service quality. This result aligns with the research model proposed by Oliveira et al. [40]. Thus, to develop the research model of the current study, Oliviera et al.’s [40] model was used as the primary source and was modified to suit the characteristics of the health sector of North Cyprus. Consequently, sub-dimensions were borrowed from the logical model of Oliviera et al. [40], including patient’s capacity to pay for services prices of healthcare services for affordability, geographic accessibility and availability of healthcare services for accessibility, and patients’ attitudes and expectations and characteristics of the healthcare services for acceptability of the quality of healthcare services in North Cyprus.

### Affordability of healthcare services

Affordability is an important concept, but it is hard to define, and this has much to do with the fact that defining affordability is a normative issue [32]. Affordability can be easily described against the backdrop of, for instance, housing [41, 42], education [43], transportation [44], utilities [45] and the healthcare industry [31]. In the case of healthcare, a product, such as medicine, or service is unaffordable when its price exceeds the total budget a person can afford. Moreover, a person should at least fulfil other basic needs after having purchased medical products or services. Therefore, good healthcare service is unaffordable if the patient, after making the purchase, does not have enough resources or money left to fulfil their basic needs [31].

### Accessibility of healthcare services

The main objective of accessibility is to improve customers’ access to healthcare services, which has always been a major concern of ministries of health in many countries [46]. The accessibility of healthcare is a multidimensional concept that can be simply defined as an individual’s ability to obtain healthcare services. Due to the non-uniform distribution of health professionals and residents, accessibility always varies across spaces.

According to the European Observatory glossary produced on health systems and policies in 1998, WHO defined availability of healthcare as calculating the proportion of people who have access to medical services. Optimal access to healthcare would conform to a situation characterized by provision of care and the timely intervention of medical staff or paramedical staff authorized in situations that require the provision of health services in the home or place in which the patient resides. At the European Union (EU) level, addressing “basic needs” and “equality” are two approaches to developing universal access. In a perfect world, all citizens would have equal access to quality healthcare services [47].

### Acceptability of healthcare services

Specific examples of different definitions, such as the terms “treatment acceptability” [48, 49] and “social acceptability” [33, 50], confirm that defining acceptability is not a straightforward matter, especially within the healthcare literature, which shows the considerable ambiguity of this concept. This shows that acceptability can be approached from an individual perspective and may also reflect a more collectively shared judgement about the nature of a treatment process. Sidani et al. [51] proposed that treatment acceptability depends on a patient’s attitude towards treatment and their judgement of its perceived acceptability before undergoing the treatment. Within this judgment judgement, factors will certainly influence the patient’s perceived acceptability, including suitability to the individual’s lifestyle and appropriateness in addressing the clinical procedure at hand. It can also be considered that perceptions of acceptability may change with the actual experience of the treatment [52]. For instance, factors such as participation in a treatment, the content of the treatment and the perceived or actual effectiveness of the treatment are likely to influence patients’ perceptions of acceptability.

### Research model and hypothesis development

**Fig. 1:**
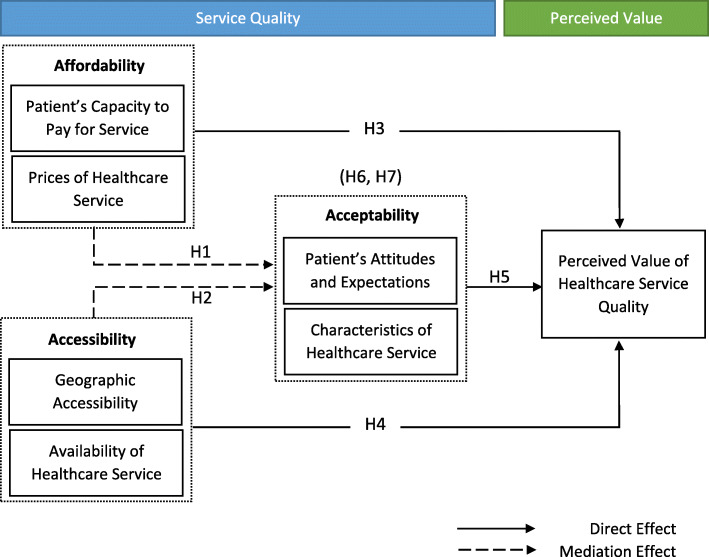
Research model (Source: [40])

Figure 1 depicts the conceptualized research model, which integrates chosen indicators from the aforementioned theoretical and empirical studies. A set of hypotheses was formulated and tested in the current study depending on the relationships between constructs.

In the hypothesized model of this study, health service quality indicators (affordability, accessibility and acceptability) play the role of independent (exogenous) variables for the perceived value of healthcare service quality as a dependent (endogenous) variable. The uniqueness of this study lies in its introduction of acceptability as a mediator of the relationship between affordability and accessibility on one side and perceived value on the other side. As the proposed structural model shows, acceptability simultaneously plays the role of the dependent variable for affordability and accessibility. Although the sub-dimensions of affordability, accessibility and acceptability were identified, the main dimensions were entered into the analysis.

#### Affordability and acceptability of healthcare services

Previous research has shown that there is a relationship between affordability and the customer’s acceptance level in the service industry, which successfully leads to an increase in profitability for an organization [36]. For example, within the food industry, an exploration of consumer affordability presented wider positive insights into consumer acceptability and market price [53]. Similar research within the transportation sector that investigated the impact of transport pricing on acceptability revealed the same acceptability level [54]. On the basis of the literature, the study hypothesized as follows:

- *H1. The affordability of healthcare services has a significant and positive effect on the acceptability of healthcare services.*

#### Accessibility and acceptability of healthcare services

Accessibility is mainly measured by availability, addressability and geographical and physical accessibility [27]. A recent qualitative study within the healthcare industry observed that even though services are accessible, they will not be accessed where the community has a low acceptance [55]. As a key factor, it can be considered that accessibility and acceptability always affect each other [29]. A two-phase qualitative study examined the relationship between accessibility and acceptability of new services among cancer patients and found that having easy access to facilities positively affects acceptability and increases patients’ willingness to use new healthcare services. Therefore, this study proposed the following hypothesis:

- *H2. The accessibility of healthcare services has a significant and positive effect on the acceptability of healthcare services.*

#### Affordability and perceived value of healthcare service quality

Affordability is a critical access component in healthcare services, especially when service costs are high and the patient has no insurance coverage [24]. However, improved affordability and financial accessibility of healthcare enhance patients’ perceived service quality [56], which is then positively correlated with the patient–provider relationship [57, 58]. Studies in India [30] and China [59] also found support for the close relationship between patients’ perceived service quality and care and perceived affordability. The findings showed a positive association between patients’ perceived value of service quality and patients’ trust in their physician, which was caused by previously reported relationships between the affordability and perceived quality of healthcare services. Supported by the literature, thus, the present study proposed the following hypothesis:

- *H3. The affordability of healthcare services has a significant and positive effect on the perceived value of healthcare service quality.*

#### Accessibility and perceived value of healthcare service quality

The perceived value of service quality is strongly affected by the accessibility of services to consumers [60, 61]. Healthcare service availability is a necessity, but it is not enough. Services should be accessible to clients if they are to be considered useful. Patients are concerned about physical and financial accessibility [28]. It has also been argued that patients show a high level of dissatisfaction because of accessibility problems with healthcare services. Lack of availability of appointments and geographical access to healthcare facilities leads to low patient satisfaction and perceived service quality ratings [35]. Measuring patient satisfaction regarding the accessibility of healthcare services can be considered a valuable predictor of perceived service quality. In addition, accessibility of care is positively associated with higher satisfaction and perceived service quality [62]. Therefore, this study proposed the following:

- *H4. The accessibility of healthcare services has a significant and positive effect on the perceived value of healthcare service quality.*

#### Acceptability and perceived value of healthcare service quality

Patients have genuine concerns about receiving acceptable healthcare [20]. Although healthcare quality frameworks contain many dimensions [48], recent modelling emphasizes the importance of “layman” views relating to acceptability. Reports have highlighted the positive role of patient acceptability rate on perceived service quality in the healthcare industry [40, 63]. Sovd et al. [26] investigated which characteristics of health service quality are most likely to determine client satisfaction with health services among adolescents, and they identified acceptability as a key determinant of client satisfaction.

Furthermore, it has been argued that socioeconomic status affects the acceptance and acceptability of service delivery [64] and influences customers’ perceived service quality [40, 63]. Authors have defined acceptance as the willingness to pay for services [65]. The willingness to pay will depend on income, but in many studies, it is assumed that the more people pay, the higher the acceptance and acceptability will be. Incentives such as lower prices, proper access and preparation, and lower insurance can stimulate customers’ acceptance or acceptability of service delivery [66]. Therefore, given the previous studies’ findings, this study hypothesized as follows:

- *H5. The acceptability of healthcare services has a significant and positive effect on the perceived value of healthcare service quality.*
- *H6. The acceptability of healthcare services has a mediating effect on the relationship between the affordability and perceived value of healthcare service quality.*
- *H7. The acceptability of healthcare services has a mediating effect on the relationship between the accessibility and perceived value of healthcare service quality.*

## Research method

### Measurement

To examine the proposed model, data were collected from respondents around the exits of public and private hospitals in North Cyprus. The survey instrument was developed and refined with the help of a group of scholars comprised of five healthcare sector specialists and academicians. Initially, 23 items were sourced from previously validated scales and adapted to the current study. In doing so, 20 relevant items for measuring healthcare service quality (affordability, accessibility and acceptability) were adopted from a survey conducted previously in the Republic of Kenya by [67] to measure the constructs in the research model. The perceived value of healthcare service quality was also measured using a three-item scale developed by He and Li [68].

Then a pilot survey was carried out among 102 respondents. Four items for service quality were later deleted in response to feedback from results of the pilot survey and the scholars. The remaining 19 questions were modified according to the aim of this study. Consequently, to maintain harmony and ensure the quality of the questionnaire, some questions were modified by the authors with minimal changes, whereas some important studies were benchmarked to modify four questions. Based on the study of Lim and Tang [9], the first question of accessibility (AS1) and the sixth question of affordability (AF6) were modified. Baltussen et al. [28] was the source of modifications to the third and fourth questions of acceptability (AP3 and AP4).

The revised 19-item questionnaire (nine items for affordability, three items for accessibility, four items for acceptability and three items for perceived value) with a five-point Likert scale, ranging from strongly disagree (1) to strongly agree (5), was employed (see Appendix A).

The questionnaire comprised two parts. The first part included questions regarding the sample’s demographic characteristics, such as gender, age, marital status, monthly income and education. The second part contained measures of the various constructs identified in the literature review, representing the four areas of affordability, acceptability, accessibility and perceived value of healthcare services.

### Sample design and data collection

Individual questionnaires were delivered and collected in person around the exits of public and private hospitals. Only people who had used the services at least once over the past 6 months were targeted. A judgmental sampling technique was applied for conducting the survey. Due to the demographic characteristics of North Cyprus, Turkish and English versions of the survey instrument were employed.

The survey took place during October and December 2019. In total, 501 questionnaires were distributed and collected, and 388 completed questionnaires were used for analysis, representing a 77.45% response rate.

Table 1 displays the sample’s demographic information including gender, age, marital status, monthly income and education.

**Table 1:** Sample characteristics (%)

Characteristic	Total
Gender	Male: 55.4%
	Female: 44.6%
Age	18–30: 31.3%
	30–50: 40.5%
	50–70: 28.2%
Marital status	Married: 48.4%
	De facto: 11.9%
	Single: 39.7%
Monthly income	Below 2000 TL: 36.5%
	2000–5000 TL: 34.3%
	Above 5000 TL: 29.2%
Education	High school: 19.9%
	College (2–4 years): 43.2%
	Graduate or above: 36.9%

## Results

SmartPLS 3.2.8, developed by Ringle et al. [69], was employed to test the hypotheses using the partial least squares-structural equation modelling (PLS-SEM) analytical approach, since it premises relatively few observations, measurement scales and few assumptions about the distribution of variables, unlike co-variance-based structural equation modelling (CB-SEM) [70, 71].

A two-stage analytical procedure was followed to test the measurement model and then the structural model [72], as this approach offers several comparative strengths over a one-step approach by allowing more robust inferences to be made as follows.

Primarily, the two-step approach provides an assessment of the significance of all pattern coefficients. Second, it allows for testing whether any structural model would show an acceptable fit. As a third advantage, one can conduct an asymptotically independent assessment of the theoretical model of interest. Lastly, the two-step approach offers a particularly valuable framework for formal assessments of the practical model of interest with the next most likely theoretical alternatives [73].

### Measurement model

Internal consistency reliability was assessed by evaluating Cronbach’s alpha (CA) and composite reliability (CR). Constructs with high internal consistency usually have highly correlated indicators. As Table 2 shows, the CA values for AF, AS, AP and PV are 0.802, 0.833, 0.815 and 0.854, respectively. Table 2 also includes the CR values for AF, AS, AP and PV, which are 0.871, 0.833, 0.759 and 0.856, respectively. Therefore, all constructs’ CA and CR values are above the suggested value of 0.7, showing internal consistency [74].

**Table 2:** Measurement model results

Construct	Item	Standardized Loadings	Cronbach’s Alpha	Composite Reliability	Average Variance Extracted	VIF	Mean	Std. Deviation
**Affordability (AF)** [Mean = 3.354, STD = 1.163]	AF1	0.637	0.802	0.871	0.573	1.988	3.041	1.228
	AF 2	0.683					3.211	1.244
	AF 3	0.769					3.513	1.098
	AF 4	0.636					3.642	1.103
	AF 5	0.728					3.650	1.144
	AF 6	0.622					3.340	1.119
	AF 7	0.820					3.569	1.077
	AF 8	–					–	–
	AF 9	–					–	–
**Accessibility (AS)** [Mean = 3.259, STD = 1.116]	AS1	0.835	0.833	0.833	0.624	1.944	3.325	1.090
	AS2	0.751					3.170	1.160
	AS3	0.783					3.273	1.098
**Acceptability (AP)** [Mean = 3.490, STD = 1.115]	AP1	–	0.815	0.759	0.512	1.533	3.348	1.198
	AP2	0.722					3.534	1.130
	AP3	0.740					3.562	1.049
	AP4	0.684					3.516	1.084
**Perceived Value (PV)** [Mean = 3.534, STD = 1.157]	PV1	0.840	0.854	0.856	0.666	2.261	3.559	1.168
	PV2	0.862					3.438	1.185
	PV3	0.741					3.603	1.117

To achieve convergent validity, the general rules of thumb are that standardized loadings should be greater than 0.6 [74] and the average variance extracted (AVE) value should be higher than 0.5 [75]. As seen in Table 2, loadings for all items exceed the benchmark, as they are 0.622 (AF6), 0.636 (AF4), 0.637 (AF1), 0.683 (AF2), 0.684 (AP4), 0.722 (AP2), 0.728 (AF5), 0.740 (AP3), 0.741 (PV3), 0.751 (AS2), 0.769 (AF3), 0.783 (AS3), 0.820 (AF7), 0.835 (AS1), 0.840 (PV1) and 0.862 (PV2), confirming that the relationships between constructs and their indicators are sufficiently strong. Only items AF8, AF9 and AP1 fail to meet the benchmark and are removed from the analysis. The AVE values are 0.573 for AF, 0.624 for AS, 0.512 for AP and 0.666 for PV. Since the values for loadings and AVE meet the threshold values recommended, it can be concluded that all constructs show sufficient evidence of convergent validity.

Discriminant validity (DV) is the degree to which the constructs employed in the model vary from each other [72]. Discriminant validity was evaluated by the Fornell-Larcker criterion [76] and Heterotrait-Monotrait ratio of correlations (HTMT) criterion [77].

The Fornell-Larcker criterion entails that the square root of the AVE for every construct should be higher than the inter-construct links. It is clear from the values in Table 3 that for a particular latent variable, the square root value of AVE is higher than the correlation value given in the corresponding row and column.

**Table 3:** Discriminant validity (Fornell-Larcker Criterion)

Construct	AF	AP	AS	PV
AF	**0.757**			
AP	0.714	**0.716**		
AS	0.712	0.700	**0.790**	
PV	0.623	0.687	0.615	**0.816**

Notes: *AF* Affordability, *AP* Acceptability, *AS* Accessibility, *PV* Perceived value of health service quality

Discriminant validity is attained, as all the HTMT values (Table 4) are below the suggested level of 0.85 [77].

**Table 4:** Heterotrait-Monotrait ratio

Construct	HTMT			
	AF	AP	AS	PV
AF				
AP	0.737			
AS	0.716	0.699		
PV	0.622	0.691	0.614	

Notes: *AF* Affordability, *AP* Acceptability, *As* Accessibility, *PV* Perceived value of health service quality

Before assessing the structural model, besides reliability and validity, multicollinearity must be checked. Following the full collinearity approach, the variance inflation factor (VIF), as advised by Kock and Lynn [78] and Kock and Hadaya [79], was used and evaluated (Table 5). This is a comprehensive procedure for simultaneously assessing both vertical and lateral collinearity. All the VIF values for the two endogenous variables (AF = 1.589 and AS = 1.589) when AP is an exogenous variable, and VIF values for three endogenous variables (AF = 1.909, AP = 1.743, AS = 1.743) when PV is an exogenous variable, are lower than the value of 5.0 recommended by Hair et al. [74], showing no sign of a collinearity issue between the latent constructs.

**Table 5:** Inner VIF values

	AF	AP	AS	PV
AF		1.589		1.909
AP				1.743
AS		1.589		1.743
PV				

Notes: *AF* Affordability, *AP* Acceptability, *AS* Accessibility, *PV* Perceived value of health service quality

A standardized root mean square residual (SRMR) value of 0.054, which is less than the suggested threshold of 0.080, implies that there are no model misspecification issues [80]. R^2^ for the acceptability of healthcare services is 0.615 and 0.517 for patient’s perceived value of healthcare service quality, suggesting that the proposed model has acceptable explanatory power [74].

### Structural model

A structural model assesses the causal relationship between constructs. As Hair et al. [72] suggested, a bootstrapping technique with re-sampling (2000 re-samples) was executed to estimate the statistical significance of the hypothesized model. The results of the hypothesized relationship testing in Table 6 provide information about all developed hypotheses. H1 shows a strong significant positive relationship between the affordability and acceptability of healthcare services (β = 0.502; *p* < 0.01; t = 5.582). H2 confirms another positive and significant relationship between the accessibility and acceptability of healthcare services (β = 0.343; *p* < 0.01; t = 3.948). The path coefficient of affordability and perceived value of healthcare service quality (0.373) is positive and significant (*p* < 0.01; t = 4.098). This result is consistent with the prediction of H3; thus, it is supported that a higher level of affordability is associated with a higher level of perceived health service quality. The reported outcomes in Table 6 also reveal that the accessibility of healthcare services has a positive and significant impact on customer’s perceived service quality (β = 0.349; *p* < 0.01; t = 3.755), which supports H4. Finally, results in Table 6 are in line with H5 and support the reasoning that the higher the level of acceptability of healthcare services, the higher their perceived service quality (β = 0.424; *p* < 0.01; t = 3.919). Therefore, H5 is also supported.

**Table 6:** Summary of hypothesis testing results

Hypotheses	Path	Estimate (Path coefficient)	t-Value	Decision
*H1*	AF → AP	0.502***	5.582	Supported
*H2*	AS → AP	0.343***	3.948	Supported
*H3*	AF → PQ	0.373***	4.098	Supported
*H4*	AS → PQ	0.349***	3.755	Supported
*H5*	AP → PQ	0.424***	3.919	Supported

Notes: *AF* Affordability, *AP* Acceptability, *AS* Accessibility, *PQ* Perceived service quality, ***p < 0.01

One of the study’s main objectives was to investigate the mediating effect of the acceptability of healthcare services in two relationships of the model: first, between affordability and perceived healthcare service quality, and second, between accessibility and perceived healthcare service quality. The mediation test was examined by conducting bootstrapping for results generated from 2000 re-samples.

As Table 7 displays, in the presence of the mediator, when the indirect effect on the relationship between AF, AP and PQ is positive and significant (β = 0.213; *p* < 0.01), there is no significant direct effect between AF and PQ. Interestingly, it is confirmed that the acceptability of healthcare services acts as a full mediator between affordability and perceived service quality of healthcare services. Similarly, in the presence of acceptability as a mediator of the relationship between AS and PQ, both the indirect (β = 0.145; *p* < 0.01) and direct (β = 0.202; *p* < 0.05) relationships remain significant simultaneously. This confirms that the acceptability of healthcare services can also partially mediate the relationship between accessibility and perceived healthcare service quality. The results in Table 7 imply the more vital role of acceptability in mediating the relationship between AF and PQ, compared with the relationship between AS and PQ.

**Table 7:** Testing of mediating effects

Hypothesis		Estimate	BC 95% CI		Mediating effects
			Lower	Upper	
*H6*	Direct effect (AF → PQ)	0.163	−0.052	0.357	Full mediation
	Indirect effect (AF → AP → PQ)	0.213***	0.105	0.392	
*H7*	Direct effect (AS → PQ)	0.202**	0.006	0.394	Partial mediation
	Indirect effect (AS → AP → PQ)	0.145***	0.060	0.277	

Notes: *n* = 388. *BC* bias corrected, *CI* confidence interval. In total, 2000 bootstrap samples; **p < 0.05, ***p < 0.01

## Discussion

Considering the pressing need to study the impact of service quality on patients’ perceived value of healthcare services in the context of North Cyprus, this study empirically analysed causal relationships between the determinants of healthcare service quality and patients’ perceived value of such services. To this end, a comparative analysis was conducted among the existing models to show why Oliveira et al. [40] was chosen as the main source for confirming the hypothesized model of this study, which includes a group of modern, effective indicators of service quality encompassing the affordability, acceptability and accessibility of healthcare services. To strengthen the model of this study by justifying it on the basis of extant literature, acceptability played the role of mediator in the model among the determinants of healthcare service quality.

This study makes significant theoretical contributions to the literature on the relationship between healthcare service quality dimensions and patients’ perceived value of healthcare services. The findings disclosed that AF was a significant and strong predictor of AP, as was hypothesized (H1). This outcome is supported by previous studies that predicted AP [36, 53, 54, 81]. As proposed in the second hypothesis (H2), this research confirmed that AS was another forecaster of AP. This finding is also in line with previous research [27, 29, 55, 82, 83]. The outcomes confirmed that AF also had a moderate and significant direct effect on the perceived value of healthcare services, as was hypothesized (H3). This finding augments the existing literature ([56–58, 59, 84]). The results also revealed that AS had a significant direct effect on PV, as was hypothesized (H4). This aligns with earlier studies ([28, 35, 84]). The final direct causal relationship between AF and PV, which was hypothesized (H5), showed a significant effect on the obtained outcomes. This finding is in alignment with former research in the field. Additionally, the study confirmed that the full mediating effect of AP on the relationship between AF and PV, stated in H6, is a unique contribution to the relevant literature. As hypothesized (H7), AP partially mediated the relationship between AS and PV, confirming the results of previous studies [20, 24–26, 30, 40, 48, 62, 66].

### Practical implications

This study contributes an explanatory model for healthcare institutions intending to measure patients’ impressions of healthcare service quality. While the purpose of this study was to investigate theoretically driven hypotheses, the findings of this research have some significant practical implications for the health system of North Cyprus.

The importance of non-financial performance dimensions, such as quality improvement, patient satisfaction and internal business procedures, has been explored since the 1990s. According to McCracken et al. [85], although the focus of health professionals conventionally was more on objective performance indicators, the findings of this research support that policymakers must consider patient satisfaction to improve the quality of health services. Focusing on the interconnection between the affordability, accessibility and acceptability of health services and health outcomes will create a competitive advantage for health managers to influence their patients’ attitudes and sustain their interest in repurchasing these services in the future.

In light of underlying theories and the findings of this research, we might deduce that when patients perceive that a hospital or clinic is ensuring their well-being by rendering more affordable and accessible medical care, this provides them with a more acceptable purchasing experience. This creates further satisfaction for the patients, which will be the source of more trust and an intention to revisit the hospital or clinic. Based on the sub-groups introduced in the research model, health providers should implement a more competitive pricing strategy in a price-sensitive society like North Cyprus based on the ability of patients to pay for medical care and medicines. Price is one of the most important decision criteria that most customers evaluate during the pre-purchasing step to choose the best option in the market. Access is another important decision-making criterion for customers to find the closest hospital with the availability of technology, knowledge and time to offer acceptable health services. There is also a crucial need for healthcare professionals in decision-making positions to understand if the characteristics of rendered services, like clinical examination and diagnosis or medical equipment, are in alignment with patients’ attitudes and meet their expectations. If they are, the patients will believe that the services are worth their money, effort and time, and this will increase their satisfaction. Accordingly, to maintain a competitive advantage, hospitals ought to implement the most recent technology and equipment to improve the quality of the offered services. Offering novel service features and fulfilling promises made to clients elevate their level of satisfaction, which, in turn, enables the provider to boost patient loyalty and future buying intention.

Governments in the Eastern Mediterranean region receive conflicting messages regarding their changing roles and responsibilities in health, particularly in relation to privatization policies and moves towards a market economy. Through ministries of health and partnerships with the private sector and non-governmental organizations, governments play an important role in healthcare development by strengthening health systems and generating human, financial and other resources. This allows health systems to achieve their goals of improving health, reducing health inequalities, securing equity in healthcare financing and responding to population needs [86].

Due to the close political, social, economic and cultural ties between North Cyprus and Turkey, the promotion of North Cyprus’s health system is, to some extent, influenced by Turkey’s health system. Perhaps, given the developing health system in North Cyprus, the localization of Turkey’s health studies results and policies could lead to more effective growth of this system. As Sahin and Ozcan [87] declared, “the government needs to focus on the efficient utilization of human resources, homogenous distribution of resources, and improve quality management programs throughout the country.”

Towards achieving the aim of this study, the Ministry of Health in North Cyprus can develop effective long-term policies to improve the main functions in hospitals and especially focus on devising a private category comprising efficient leadership, ease of financing, attraction of local and foreign investors, assistance in providing technologies and facilities, generation of inputs for health development, such as human resources for health, and biomedical technology.

Last but not least, as Kilbourne’s [88] study showed, a positive and consistent interrelation exists between health insurance coverage and healthcare service outcomes. It also discussed the connection between health insurance and more favourable use of healthcare services and better experiences for patients. Thus, the government can play a vital role by providing more effective health insurance policy programs to ensure that people obtain the healthcare services they need without suffering financial hardship when paying for them. This will improve access to healthcare considerably and diminish the adverse effects of having been uninsured.

So long as the policies target making healthcare services more affordable and accessible, we can expect to decrease the gap between patients’ expectations and the healthcare services’ performance.

### Limitations of the study and future directions

As with all studies, the current study faced some limitations which can provide avenues for future researchers in the field. First, as the data were gathered from the Turkish Republic of Northern Cyprus only, the generalizability of the findings is constrained. Thus, future studies can replicate the model and choose different populations from other parts of the world. Another suggestion is conducting a comparative study which can be extended by analysing how patients’ perceptions of the modelled variables differ in two or more population sets. Finally, it should be noted that we collected the data from the health service industry in North Cyprus just before the outbreak of the COVID-19 pandemic. However, during the pandemic, societies have struggled with many unexpected social, economic, political and cultural troubles. Further studies on the effect of the global health crisis on the model’s predictive performance seem indispensable.

## Conclusion

This study highlighted relationships among the determinants for assessing service quality and patients’ perceived value of healthcare services. To achieve reliable healthcare service quality enhancement, the essential modern components of health service quality should be known. As the findings of Kruk and Freedman [89] also confirmed, this issue is important for developing countries like North Cyprus, whose health system is under reform.

According to the outcomes of this research, in North Cyprus, a more affordable and accessible health service will improve the value perceived by patients by increasing the level of acceptability of the services. Based on the sub-dimensions of healthcare service quality identified in the research model, patients’ capacity to pay for healthcare services should be one of the most important price-determining factors to make the services more affordable. It is also advisable for health professionals in North Cyprus to ensure better geographic accessibility and availability of healthcare services to improve the characteristics of the services and reduce health inequalities to increase patient satisfaction. To the best of our knowledge, this study is one of the first to examine the relationship between the modern dimensions of healthcare service quality (affordability, accessibility and acceptability) and patients’ perceived value of these services in North Cyprus. In sum, in line with the findings of Braithwaite et al. [90], modern performance measures ensure that healthcare institutions understand patients’ perceptions and can translate this as part of their organization’s vision.

## Data Availability

The data were collected by questionnaire and recorded anonymously and confidentially. However, it is available to share for reviewing process.

## Appendix A

**Table 8:** Research Questionnaire

Construct	Item	
*Affordability*		
Patient’s Capacity of Paying for Services	AF1	My hospital’s managers take into account the customer’s ability to pay when they decide for price of services and medicines.
	AF2	When I am using a medical care service, I always ask for the one with the lowest price.
	AF3	When I am using a medical care service in my hospital, I believe it will be worth my money.
	AF4	It is easy for me to afford the costs of medical care and medicines because my hospital generally accepts most major health insurers.
	AF5	My hospital has an advantage for me to pay the bill during the longer period.
Prices of Healthcare Services	AF6	The health services are affordably priced in my hospitals.
	AF7	It is easy for me to find out how much the healthcare services will cost in my hospitals.
	AF8	Two similar healthcare services sold at different prices in my hospitals and other hospitals.
	AF9	Usually, I can buy the healthcare services we need provided by my hospital, because of the affordable prices.
*Accessibility*		
Geographic Accessibility	AS1	The location of my hospital is easily accessible.
Availability of Healthcare Services	AS2	I have confidence that high quality medical care is available in my hospital for me and my family.
	AS3	The health care services I usually need are available all the time in my hospital.
*Acceptability*		
Patient’s Attitudes and Expectations	AP1	There are hospitals in my neighborhood where I should never buy health services because they sell low-quality services.
	AP2	The healthcare services rendered in my hospital meet my expectations because of the good quality.
Characteristics of Healthcare Services	AP3	In my hospital, acceptable medical services are provided because of good clinical examination and diagnosis.
	AP4	High-quality healthcare services are offered in my hospital because of adequacy of medical equipment.
*Perceived value of healthcare services quality*		
	PV1	Comparing with major competitors, my hospital is a good choice.
	PV2	I would say that my hospital provides superior service.
	PV3	I believe my hospital offers excellent service.

## Abbreviations

WHO: World Health Organization
UHC: Universal health coverage
EU: European union
PLS: Partial least squares
SEM: Structural equation modeling
VIF: Variance inflation factor
CR: Composite reliability
AVE: Average variance extracted
AF: Affordability of healthcare services.
AS: Accessibility of healthcare services
AP: Acceptability of healthcare services
PV: Perceived value of healthcare services quality
DV: Discriminant validity
HTMT: Heterotrait- monotrait ratio
SRMR: Standardized root mean square residual
BC: Bias corrected
CI: Confidence interval
WOM: Word-of-mouth
